# Low-Intensity CD66c Expression Orchestrates an Immunosuppressive Niche Promoting Residual Disease in Pediatric ProB Acute Lymphoblastic Leukemia

**DOI:** 10.3390/cells15050437

**Published:** 2026-02-28

**Authors:** Gabriela Zamora-Herrera, Rubí Romo-Rodríguez, Jebea A. López-Blanco, Laura Alfaro-Hernández, Diana Casique-Aguirre, Juan Carlos Núñez-Enríquez, Michael Schnoor, Dalia Ramírez-Ramírez, Rosana Pelayo

**Affiliations:** 1 Laboratory of Oncoimmunology and Cytomics of Childhood Cancer, Centro de Investigación Biomédica de Oriente, Instituto Mexicano del Seguro Social, Puebla 74360, Mexico; gabriela.zamorah@alumno.buap.mx (G.Z.-H.); rubi.romo@imss.gob.mx (R.R.-R.); jebea22@yahoo.com.mx (J.A.L.-B.); laura.alfarohernandez@viep.com.mx (L.A.-H.); dianacasique@gmail.com (D.C.-A.); 2 Instituto de Fisiología, Benemérita Universidad Autónoma de Puebla, Puebla 72570, Mexico; 3 Secretaría de Ciencia, Humanidades, Tecnología e Innovación (Secihti), Mexico City 03940, Mexico; 4 Facultad de Ciencias Químicas, Benemérita Universidad Autónoma de Puebla, Puebla 72570, Mexico; 5 División de Investigación en Salud, Unidad Médica de Alta Especialidad (UMAE) Hospital de Pediatría “Silvestre Frenk Freund” Centro Médico Nacional Siglo XXI, Instituto Mexicano del Seguro Social, Mexico City 06720, Mexico; jcarlos_nu@hotmail.com; 6 Department of Molecular Biomedicine, Cinvestav-IPN, Mexico City 07360, Mexico; mschnoor@cinvestav.mx; 7 Unidad de Educación e Investigación, Instituto Mexicano del Seguro Social, Mexico City 06725, Mexico

**Keywords:** childhood acute lymphoblastic leukemia, tumor microenvironment, immune contexture, CD66c, risk stratification

## Abstract

**Highlights:**

**What are the main findings?**

- ProB-ALL blasts with low-intensity CD66c expression exhibit a “molecular stealth” phenotype associated with a significantly higher risk of detectability of Measurable Residual Disease and mortality.
- Leukemic CD66c^low^ cells orchestrate a bi-directional remodeling of the bone marrow niche.

**What are the implications of the main findings?**

- Beyond binary (positive/negative) CD66c assessment leading to intensity-based immunophenotypic substratification is essential for identifying hidden chemoresistant reservoirs at diagnosis.
- The low immunogenicity CD66c^low^ blast population identifies high-risk patients who would otherwise be misclassified by conventional cytometric thresholds.

**Abstract:**

**Background/Objectives:** B-cell precursor acute lymphoblastic leukemia (B-ALL), the most common pediatric acute leukemia (AL), is frequently characterized by aberrant antigen expression, which aids diagnosis and prognosis. The myeloid antigen CD66c is notably frequent in B-ALL and has been proposed as a marker of disease aggressiveness and treatment response. Evaluating CD66c in Mexican pediatric patients may provide insights into disease biology. **Methods:** A cohort of 128 pediatric patients was referred to the Laboratory of Oncoimmunology and Cytomics of Childhood Cancer (OCL) at Instituto Mexicano del Seguro Social (IMSS) for immunophenotyping tests between March 2022 and November 2023. Additionally, control bone marrow (BM) samples were assessed. Aberrant antigen expression in hematopoietic populations and BM microenvironment stroma phenotyping were performed. **Results:** In total, 84.38% of B-ALL patients exhibited aberrant expression of ≥1 myeloid antigen. Among CD66c-positive patients, 13.79% had detectable measurable residual disease (MRD) during follow-up and 20.69% died. Mesenchymal stromal cells (MSCs) from patients with positive or low CD66c expression displayed inflammatory profiles. ProB leukemias with low CD66c expression were more likely to exhibit detectable MRD, increased mortality, and reduced survival. **Conclusions:** Low CD66c expression induces molecular stealth that could favor immune evasion and niche persistence, thereby increasing the risk of relapse and therapeutic failure.

## 1. Introduction

B-ALL is the most common subtype of AL in pediatric patients under 19 years of age, accounting for more than 75% of AL cases [1,2]. B-ALL develops by malignant transformation of immature lymphocyte precursors in the bone marrow (BM) [3]. Immunophenotypic profiles of leukemic cells are compared with those of normal hematopoietic cells to determine lineage assignment and maturational stage, as well as to identify aberrant phenotypes. Many antigens have been reported to be aberrantly expressed in B-ALL cases in association with specific recurrent molecular abnormalities [4]. Aberrant expression is defined as the presence of an antigen belonging to some other lineage and has been widely studied as a prognostic indicator and as a marker of residual disease [5]. The regulatory mechanisms associated with aberrant markers suggest a possible lineage indecisiveness or poor genetic programming [6].

The myeloid antigen CD66c (Carcinoembryonic antigen (CEA)-related cell adhesion molecule 6, CEACAM6, previously called Nonspecific crossreacting antigen, NCA 90/50 and KOR-SA3544 antigen) is frequently expressed in B-ALL, and is a heavily glycosylated glycosylphosphatidylinositol (GPI)-anchored protein belonging to the carcinoembryonic antigen family, having two constant Ig-like domains and one variable Ig-like domain. This cell adhesion protein is generally upregulated in pancreatic adenocarcinoma, breast cancer, lung cancer, gastric cancer, colon cancer and oral squamous cell carcinoma; its expression is associated with the promotion of tumor progression, invasion and metastasis [7,8,9,10,11,12]. The expression of CD66c is observed only in granulocytes and its precursors among normal hematopoiesis. Its expression in B-ALL has been associated with *BCR::ABL* positivity, hyperdiploidy and *ETV6::RUNX1* negativity [13,14].

The significance of CD66c is of particular interest given its reported association with disease aggressiveness and treatment response. Here, we performed the first comprehensive analysis of aberrant CD66c expression in Mexican pediatric patients diagnosed with B-ALL recruited from five hospitals located in Puebla, Oaxaca and Tlaxcala.

## 2. Materials and Methods

### 2.1. Patient Characteristics and Sample Collection

This study was conducted in accordance with the Declaration of Helsinki and was approved by the Ethics, Research and Biosafety Committees of the National Committee of Scientific Research at IMSS (R-2020-785-177 and R-2023-785-047). Informed consent was obtained from parents before collecting any samples. A total of 128 patients younger than 18 years of age and diagnosed with B-ALL who were referred to the OCL at IMSS for immunophenotyping tests between March 2022 and November 2023 were included in the study. This cohort consisted of pediatric patients treated at various healthcare institutions, including Hospital de la Niñez Oaxaqueña (Oaxaca, México), Hospital Infantil de Tlaxcala (Tlaxcala, México), Hospital del Niño Poblano and Hospital de Especialidades Centro Médico Nacional General de Div. “Manuel Ávila Camacho, IMSS” (Puebla, México). Additionally, phenotypic patterns of normal hematopoietic cells were collected from 4 non-AL BM samples from individuals (patients younger than 18 years of age initially evaluated for suspected leukemia who, upon immunophenotyping test, showed no blast cells and demonstrated normal B-cell maturation patterns) and 6 healthy BM samples from individuals (patients younger than 18 years of age who sustained accidental injuries and receive care at the Hospital de Traumatología y Ortopedia, IMSS, Puebla) were included to establish reference intervals and mean fluorescence intensity (MFI) values of hematopoietic populations, enabling comparisons with normal counterparts. BM specimens were obtained by aspiration prior to any treatment for diagnostic purposes and again at the end of induction therapy (EOI) to monitor treatment response, performed at a median of 56 days, to monitor treatment response following both international and institutional guidelines.

### 2.2. Chemotherapy Protocol

All participating hospitals adhered to a chemotherapy protocol for B-ALL based on the St. Jude Total XV regimen, which is structured into three sequential phases: Remission Induction (42 days), Consolidation (8 weeks), and Maintenance (120 weeks), the latter incorporating two risk-adapted reinduction blocks. The Remission Induction phase is based on the four cornerstone drugs historically used in ALL treatment: prednisone (40 mg/m^2^/day orally), vincristine (1.5 mg/m^2^ weekly for four doses), daunorubicin (25 mg/m^2^ weekly for two doses), and L-asparaginase (10,000 IU/m^2^ every 48 h for 6 to 9 doses). This is followed by Induction Phase B, involving three drugs: cyclophosphamide (1 g/m^2^ single dose), cytarabine (75 mg/m^2^/day for 8 doses), and 6-mercaptopurine (60 mg/m^2^/day for 14 days). The Consolidation phase consists of high-dose methotrexate tailored to the patient’s assigned risk category: 2.5 g/m^2^ for low-risk and 5 g/m^2^ for standard- and high-risk patients, administered every 14 days for four doses. This is accompanied by CNS-directed intensification with four doses of intrathecal chemotherapy and daily 6-mercaptopurine (50 mg/m^2^/day). The Maintenance phase spans 120 weeks and includes multidrug therapy, featuring two risk-adapted reinduction blocks during weeks 7–9 and 17–19, respectively.

### 2.3. Classification of B-ALL and Assessment of Treatment Response

BM samples were processed and stained following EuroFlow guidelines. Initially, the Acute Leukemia Orientation Tube (ALOT) was used to identify leukemia cases. In samples exhibiting CD19 and cyCD79a expression, the B-ALL complementary antibody panel was subsequently applied. B-ALL cases were classified into the following subtypes: ProB-ALL (CD34^+^ CD19^+^ cyCD79a^+^), ProB-PreB-ALL (CD34^−/+^ CD19^+^ cyCD79a^+^) and PreB-ALL (CD34^−^ CD19^+^ cyCD79a^+^). For follow-up, a MRD test was performed, BM samples from B-ALL patients were processed according to the EuroFlow bulk lysis standard operating procedures, followed by staining with the B-ALL MRD antibody panel. For MRD assessment, at least 5 million viable cells were analyzed, and were classified as detectable when blast cells accounted for ≥0.01% of them. Sample acquisition was conducted using BD FACSLyric or BD FACSCanto II cytometers (Becton, Dickinson and Company, San Jose, CA, USA). Flow cytometry data analysis was performed using Infinicyt 2.0.4.a.013 software.

### 2.4. Aberrant Expression Markers in B-ALL

Aberrant marker expression in blast cells was considered positive (classified as low or positive) for myeloid and immature markers (CD66c, CD33, CD13, CD15, and CD123) when the MFI of the blast population exceeded that of the reference negative population (T lymphocytes) and was compared with the corresponding reference positive population (neutrophils for CD66c, CD13 and CD15; monocytes for CD33; and normal B-lymphocyte precursors for CD123).

### 2.5. Isolation and Immunophenotyping of BM MSCs

MSCs were isolated through adhesion and morphology. Subsequent flow cytometry analyses were conducted in accordance with procedures outlined in our previous publication [2].

### 2.6. Subpopulation Clustering and Immunological Landscape

To characterize the immunohematopoietic landscape and clonal heterogeneity in B-ALL, particularly within the ProB subtype aberrantly expressing myeloid antigens, we performed high-dimensional flow cytometry analysis followed by unsupervised clustering using the Phenograph algorithm implemented in FlowJo. Leukemic and immune cell populations were identified based on surface marker expressions, including CD66c, CD123, and CD33, which were used to delineate aberrant myeloid phenotypes. Uniform Manifold Approximation and Projection (UMAP) dimensionality reduction was applied to visualize phenotypic substructures and antigen expression patterns across diagnostic and MRD timepoints. Neutrophils, lymphocytes, and blasts were annotated according to canonical markers and visualized in distinct color-coded clusters. Heatmaps of CD66c expression were overlaid on UMAP plots to assess its distribution within leukemic and immune compartments. Comparative analyses were conducted between ProB cases with isolated CD66c expression and those co-expressing CD123 and/or CD33, enabling stratification of immunophenotypic profiles and assessment of reconstitution dynamics during induction therapy.

### 2.7. Crude Mortality Analysis by CEACAM6 Expression Group in the TARGET-ALL Phase II Cohort

RNA-Seq gene expression data (RPKM) and clinical variables from pediatric patients with B-ALL were obtained from the TARGET-ALL Phase II project. CEACAM6 expression data were retrieved from the TOIL/XenaBrowser repository, while clinical data was sourced from the GDC Data Portal. Only samples obtained at the time of initial diagnosis (GDC sample type code “−03”) were included in this analysis (*n* = 39 patients with complete expression and vital status data), thereby excluding recurrent tumor samples (“−04”), blood-derived normal/germline control samples (“−09/−10”), and recurrent peripheral blood samples (“−40”). Crude mortality (vital status at last follow-up, OS_STATUS, available for all 39 patients) was used as the primary descriptive outcome, since overall survival in months (OS_MONTHS) was available for only 16 of the 39 patients. CEACAM6 expression was stratified into three descriptive groups using a natural-gap approach: expression thresholds were defined from empirically observed discontinuities in the ordered RPKM distribution of the 39 diagnosis-stage patients, identified without reference to clinical outcomes. The Negative/Low boundary corresponds to the largest gap among values below 5 RPKM (gap = 1.2232 RPKM units, located between 1.9334 and 3.1566 RPKM), and the Low/Positive boundary corresponds to the second-largest gap in the full ordered distribution (gap = 4.6782 RPKM units, located between 4.3403 and 9.0185 RPKM), yielding Negative (RPKM ≤ 1.9334, *n* = 31), Low (1.9334 < RPKM ≤ 9.0185, *n* = 4), and Positive (RPKM > 9.0185, *n* = 4) groups. As a pre-specified sensitivity analysis, an unsupervised equal-size (tertile) stratification and Jenks natural breaks of the same cohort were also examined.

### 2.8. Statistical Analysis of Crude Mortality

Crude mortality rates (percentage of patients deceased at last follow-up) were calculated for each CEACAM6 expression group (Negative, Low, Positive) as defined in Section 2.7. Pairwise comparisons of crude mortality rates between groups were performed using Fisher’s exact test. All analyses were implemented in Python 3.11 using pandas, numpy, scipy, matplotlib, and seaborn. Bar charts were generated with standardized parameters.

### 2.9. Statistical Analysis

Bar graphs represent mean with standard deviation (SD). GraphPad Prism version 10.1.1 for Windows (La Jolla, CA, USA) was used for data analysis. Within-group differences were determined using the non-parametric Kruskal–Wallis test with Dunn’s post-test for comparing continuous variables. Relative risk values of detectable MRD (RR_MRDd_) and 95% confidence interval (CI) were calculated with Koopman asymptotic score, stratified by CD66c expression levels (comparing each group to the CD66c-negative group). Statistically significant differences were considered at *p* values < 0.05.

## 3. Results

### 3.1. Patient Cohort Characteristics

This study included a cohort of 138 pediatric patients, divided into three groups. The first group corresponds to patients diagnosed with B-ALL (Table 1, *n* = 128), aged 1 to 17 years (mean 8.15 ± 4.78), of whom 51.56% were male. At the time of clinical diagnosis, patients were classified as follows: 50.78% ProB-ALL, 34.38% ProB-PreB-ALL, and 14.84% PreB-ALL. The other groups consisted of control children: four non-AL patients and six healthy individuals. In the non-AL group, all patients (100%) were male, with a mean age of 2.88 years. In the healthy control group, 83.33% were male, with a mean age of 12.83 years (Table 1).

### 3.2. Impact of Aberrant Marker Expression on Treatment Response in B-ALL

**Figure 1:**
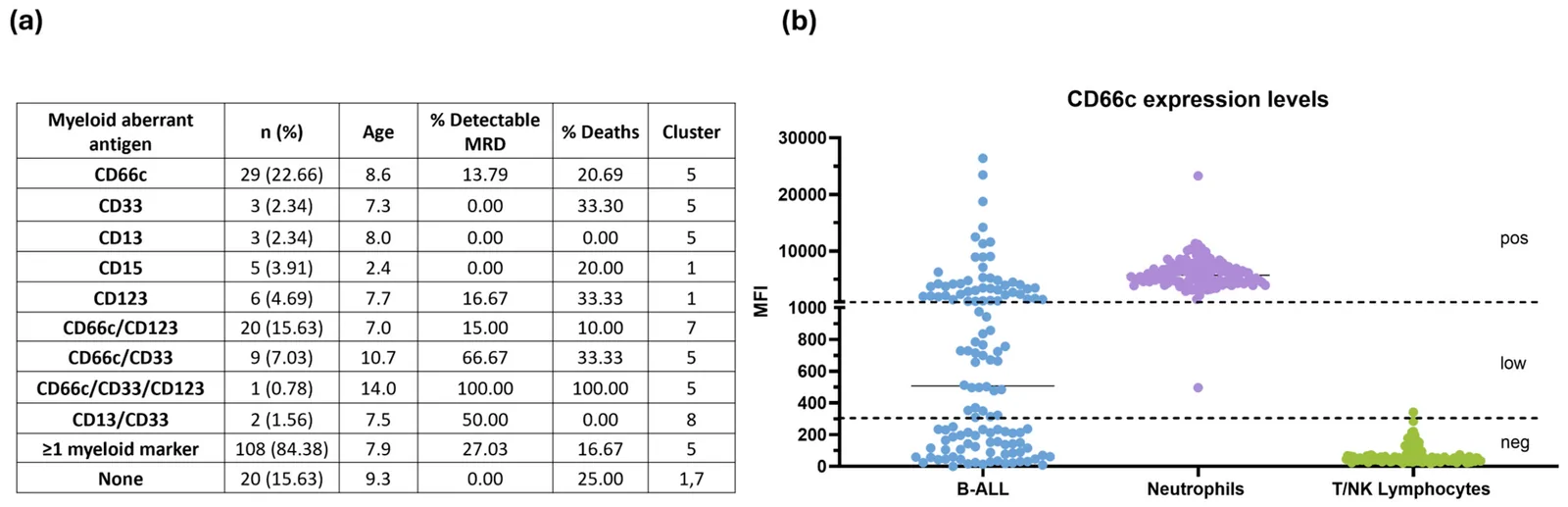
**Myeloid antigens are commonly aberrantly expressed in blasts of pediatric B-ALL.** (**a**) Clinical features and their association with the expression of aberrant myeloid markers in pediatric B-ALL. (**b**) CD66c expression levels. Neutrophils were used as a positive control for CD66c expression, whereas T/NK lymphocytes were used as a negative control for CD66c expression, mean fluorescence intensity (MFI) is shown. B-ALL = 128; Neutrophils = 128; T/NK Lymphocytes = 126. Pos = positive, neg = negative.

After defining the expression patterns of aberrant myeloid and immature markers in our B-ALL group (Supplementary Figure S1), we evaluated their association with susceptibility to adverse events (Figure 1a). MRD detectability and mortality were used as outcome measures. Within the B-ALL group, only 15.63% of patients showed no aberrant marker expression. In contrast, 84.38% exhibited aberrant expression of ≥1 myeloid antigen, with CD66c and CD123 being the most frequent.

CD66c was expressed in 22.66% of B-ALL patients, representing the most prevalent individual aberrant marker. Among patients with CD66c expression, 13.79% had detectable MRD during follow-up and 20.69% died. Previously, our group identified, through multidimensional and integrated analyses, distinct patient clusters associated with higher risk; notably, cluster 5 displayed an immunophenotypic profile linked to poorer prognosis. Consistent with these findings, we observed an increased prevalence of detectable MRD and mortality among patients classified within cluster 5 and those expressing CD66c.

### 3.3. Substratification of B-ALL Based on CD66c Expression

Given the high prevalence of CD66c expression on blast cells at diagnosis and the need to refine risk-stratification for B-ALL patients, we classified the cases as negative, low or positive based on the MFI comparison between B-ALL blast cells, neutrophils (positive control) and lymphocytes (negative control) (Figure 1b).

**Figure 2:**
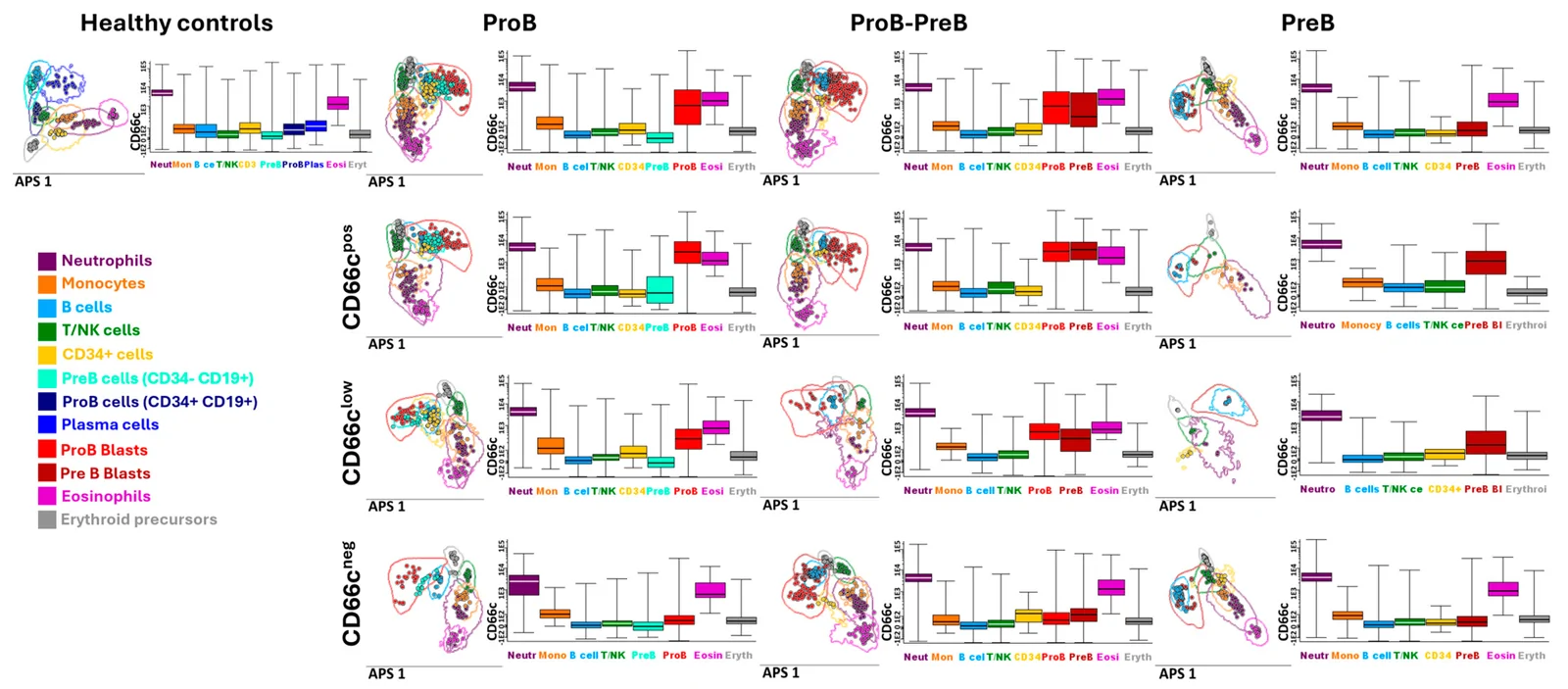
**Substratification of B-ALL based on CD66c expression.** Bars represent CD66c expression in immune cells, erythroid precursors and blasts according to B-ALL subtype and healthy controls. Profiles were performed based on automatic population separator (APS). ProB = 65; ProB-PreB = 44; PreB = 19; Healthy controls = 10.

B-ALL subtypes were further substratified based on CD66c expression in blasts, enabling the identification of distinct immunotumoral profiles in leukemias with aberrant marker expression (Figure 2).

Notably, ProB-subtype leukemias exhibited a higher frequency of positive and low-level CD66c expressions. Conversely, ProB-PreB leukemias predominantly displayed either positive expression or absence of this aberrant marker, while PreB leukemias showed minimal CD66c expression.

Furthermore, immunotumoral profiles provided by Automatic Population Separator (APS), a tool based on Principal Component Analysis, revealed distinct differentiation between ProB and ProB-PreB leukemias expressing low CD66c levels and those with positive expression. This observation is significant as it may indicate immune system reconfiguration at disease onset, potentially impacting prognosis. The heterogeneity observed in immunohematopoietic systems highlights the possibility of differential tumor behavior that could influence disease progression.

### 3.4. CD66c Aberrancy Shapes the Stromal Niche

**Figure 3:**
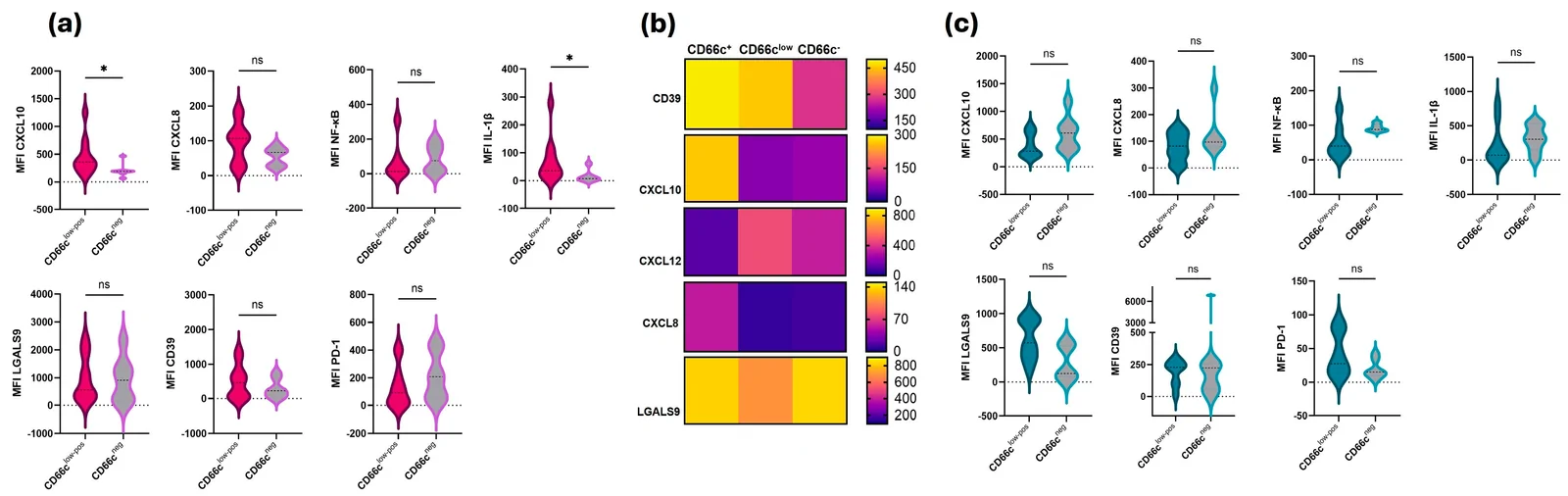
**Impact of aberrant CD66c expression on the Mesenchymal Stromal Cell Microenvironment in B-ALL.** Evaluation of inflammatory and suppressive profiles in MSCs derived from patients with B-ALL. (**a**) Phenotypic assessment of MSCs obtained at disease diagnosis (*n* = 18) according to the presence or absence of blasts with aberrant CD66c expression. (**b**) Stromal niche microenvironment profile based on the expression of CD39, CXCL10, CXCL12, CXCL8, and LGALS9 in MSCs at diagnosis, according to CD66c levels (CD66c^pos^ = 7, CD66c^low^ = 4, CD66c^neg^ = 7). (**c**) Phenotypic evaluation of MSCs obtained during disease follow-up (*n* = 16). CXCL, C-X-C motif chemokine ligand; LGALS9, galectin-9; MFI, Median Fluorescence Intensity; * *p* < 0.05. ns, not significant. Graphs include violin plots and box-and-whisker plots. The heatmap displays ΔMFI means, obtained by subtracting the MFI of the staining from the sample autofluorescence MFI.

Since ALL is generated and expanded in BM niches, we wanted to explore phenotypic characteristics of primary MSCs which are key players in BM niches at normality and disease. Eighteen MSCs cultures derived at diagnosis and sixteen during disease follow-up time were assessed. At diagnosis, MSCs from patients whose blasts exhibited positive or low CD66c aberrant expression tended to display inflammatory profiles (Figure 3a), characterized by a significant increase in CXCL10 and IL-1β compared with patients without CD66c aberrancy.

To better distinguish the microenvironmental-stromal profile of patients with CD66c aberrancy, a heatmap was generated for the CD66c^pos^, CD66c^low^, and CD66c^neg^ groups. Although the first two groups share the aberrancy, their microenvironmental profiles differ. CXCL10 appears to be a substantial difference between them, as the CD66c^pos^ group is the only one showing high expression of this chemokine ligand. A similar pattern is observed for CXCL8, but to a lesser extent. Conversely, high expression of LGALS9 was detected across all groups, and CD39 exhibited elevated expression in the stromal cells of patients with CD66c aberrancy (Figure 3b). In the case of MSCs derived from follow-up disease samples, a tendency toward increased LGALS9 expression was observed in patients with the aberrancy (Figure 3c).

To further assess whether this pattern was maintained when considering only ProB-ALL cases, the behavior was confirmed. Minor variations were observed, but the overall trend persisted (Supplementary Figure S2).

### 3.5. Hematopoietic Reconstitution in ProB Leukemias with Aberrant Expression of CD66c Shows Alterations in Remission Induction

**Figure 4:**
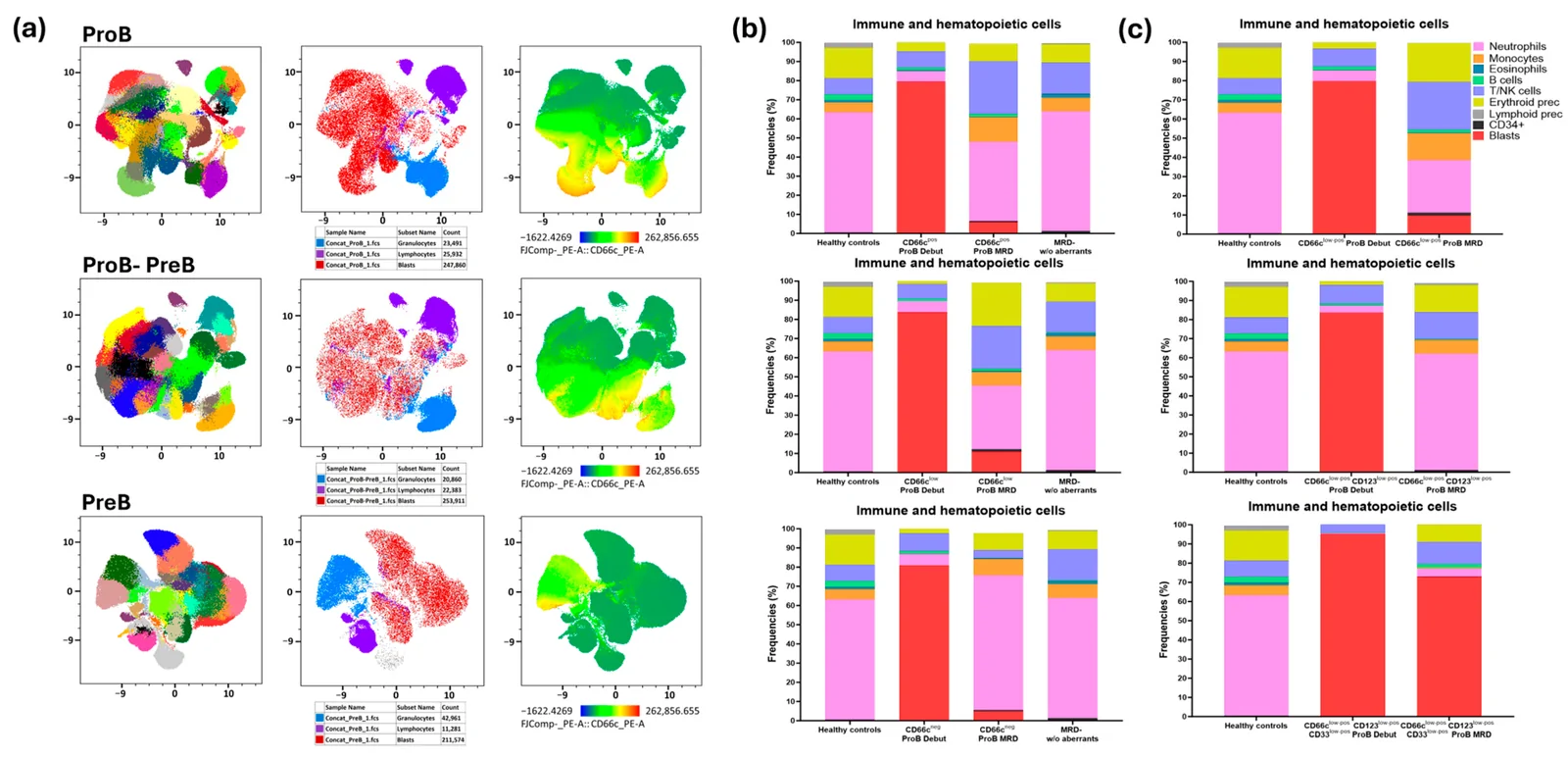
**Clonal heterogeneity and immunohematopoietic system reconstitution during response to induction therapy differ in ProB leukemias aberrantly expressing CD66c antigen.** (**a**) Phenotypic subpopulations of immunotumoral cells in different leukemia subtypes. Phenograph analysis is shown in the left panel; granulocytes are shown in blue, lymphocytes in purple, and blasts in red in the middle panel; CD66c expression is observed in the heatmap of the UMAP (right panel). (**b**) Immunohematopoietic populations in the ProB leukemia subtype, both at diagnosis and in MRD, with different levels of CD66c antigen expression. MRD-w/o aberrants = Undetectable MRD without aberrant antigens at the diagnosis. (**c**) Immunohematopoietic populations at initial diagnosis and in MRD of ProB-ALL aberrantly expressing CD66c (upper panel), CD66c/CD123 (middle panel), and CD66c/CD123/CD33 (lower panel).

Since the immunotumoral context plays a key role in disease prognosis, the immunohematopoietic profiles of the different B-ALL subtypes were characterized at onset. The results revealed that ProB leukemias exhibit greater clonal heterogeneity associated with low CD66c expression, compared to ProB-PreB and PreB leukemias, which show lower clonal diversity (Figure 4a). The UMAP heatmap shows that ProB leukemias exhibit higher levels of CD66c expression and a greater number of phenotypically distinct clones, in contrast to the other B-ALL subtypes.

Furthermore, monitoring of MRD indicated that patients with ProB leukemias, who presented with low CD66c levels at onset, showed a significant decrease in BM hematopoietic reconstitution (Figure 4b). This effect is intensified when low CD66c expression is combined with myeloid coexpressions, such as CD33 and CD123, resulting in markedly compromised immune regeneration (Figure 4c). These findings suggest that low CD66c expression in ProB leukemias could confer an advantage for tumor establishment, favoring disease development over normal hematopoiesis.

### 3.6. Low CD66c Expression on ProB-ALL Blast Cells Is Associated with Increased MRD Detectability, Reduced Overall Survival and a Higher Mortality Rate

**Figure 5:**
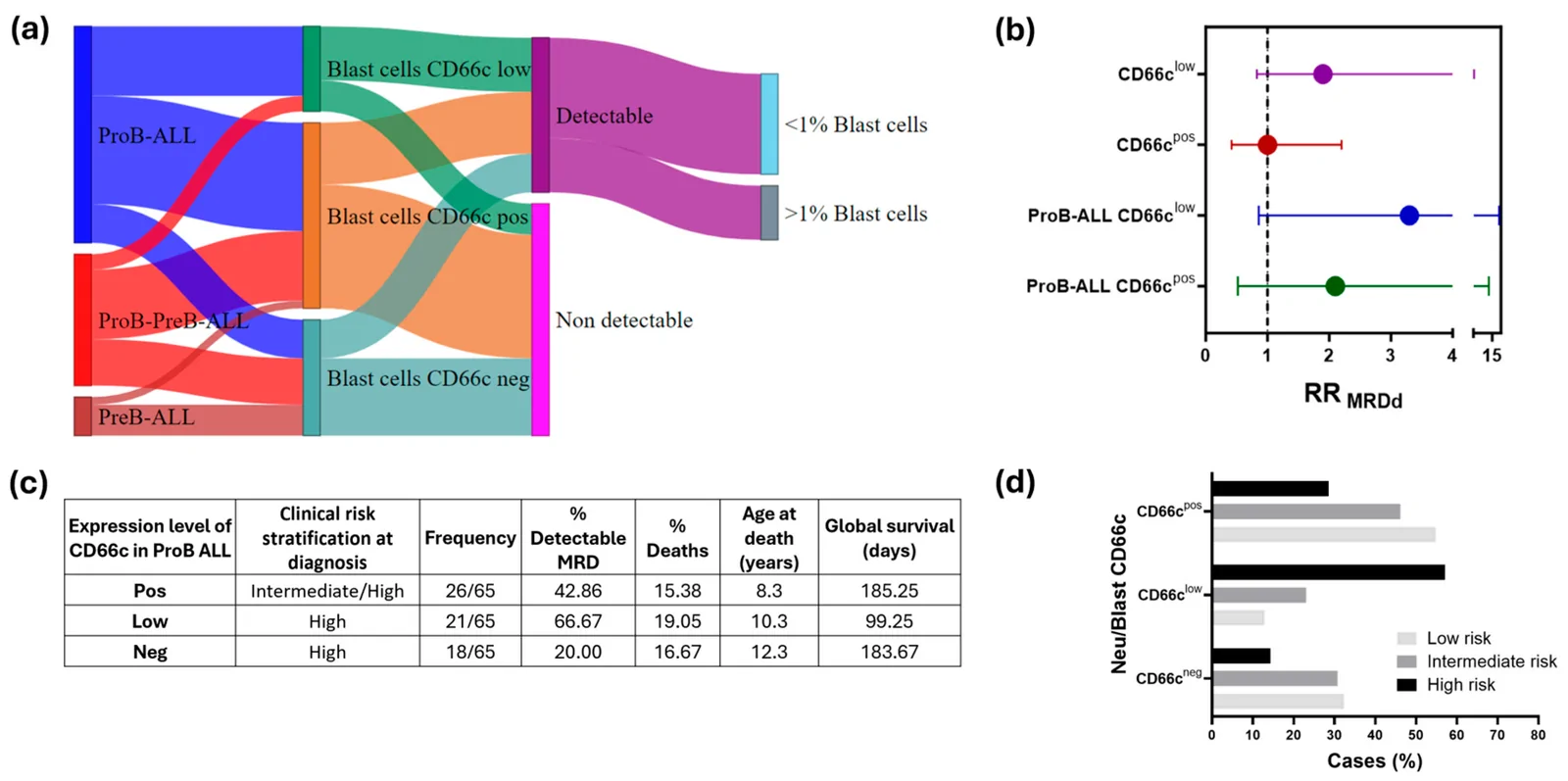
**Low CD66c expression on ProB-ALL blast cells are associated with increased MRD detectability, reduced overall survival, and a higher mortality rate.** CD66c expression levels were categorized as positive, low or negative based on the comparison of the MFI between blast cells and neutrophils, which served as a positive control for expression. (**a**) Sankey diagram compares the number of cases across B-ALL subtypes, their CD66c blast expression, and MRD outcomes, along with their respective frequencies. (**b**) Relative risk values of detectable MRD (RR_MRDd_) were calculated based on B-ALL CD66c expression. (**c**) Comparison of MRD detectability, mortality rates, age at death, and overall survival in the ProB-ALL subtype based on CD66c expression. Global survival was calculated from the beginning of the treatment to the last follow-up or death. (**d**) Risk stratification at the end of induction, using MRD and its relationship with the Neutrophil/blast CD66c ratio (CD66c^pos^, CD66c^low^, CD66c^neg^). Considering MRD, patients were stratified into three categories: non-detectable MRD (ND; below the detection limit), intermediate risk (IR; <1% blasts), and high risk (HR; ≥1% blasts).

To identify a high-risk group within the B-ALL cohort based on CD66c expression on blast cells, we analyzed each B-ALL subtype, assessing CD66c expression levels and their association with MRD detectability, frequency of deaths and global survival. First, we visualized the distribution of cases in a Sankey plot (Figure 5a), illustrating the relationship between B-ALL subtypes, CD66c expression levels, MRD detectability, and, within the MRD detectable group, the proportion of cases exceeding a 1% residual blast cell cut off. Notably, a few cases with negative CD66c expression exhibited detectable MRD. Next, we calculated the relative risk of MRD detectability (RR_MRDd_) (Figure 5b and Supplementary Table S1). In the overall B-ALL cohort, we observed a trend toward increased MRD risk in patients with low CD66c expression; however, this association did not achieve statistical significance, whereas no risk was detected in the CD66c-positive group in comparison with the CD66c-negative group. Further analysis within the ProB-ALL subgroup revealed that blast cells with low CD66c expression were more likely to exhibit residual disease compared to those with negative expression, suggesting a 3.3-fold increased risk of detectable MRD. Additionally, ProB-ALL cases with low CD66c expression demonstrated a higher frequency of deaths and reduced overall survival (OS) (Figure 5b).

### 3.7. Association Between CD66c Expression Levels and Risk Stratification During Remission Induction

Considering the potential role of CD66c in creating an immunosuppressive microenvironment and its involvement in B-ALL subtypes, particularly ProB CD34^+^, we examined the expression of this marker and its correlation with MRD during remission induction.

Patients exhibiting CD66c^pos^ phenotypes demonstrated improved responses to chemotherapy, with 54.8% (17/31) of individuals classified as having undetectable or low-risk MRD (MRD-LR). Conversely, a higher proportion of patients reclassified as high risk (MRD-HR), characterized by ≥1% residual blasts in BM during remission induction, displayed CD66c^low^ phenotypes, accounting for 57.1% (4/7) of this high-risk group (Figure 5d).

A statistically significant difference was observed between CD66c^pos^ and CD66c^low^ phenotypes considering the number of patients classified as MRD-HR (*p* = 0.04). However, no distinctions were found among patients with negative CD66c expression across the three risk groups.

### 3.8. CEACAM6 Expression in the TARGET-ALL Phase II Cohort: A Preliminary Exploratory Characterization of Crude Mortality

**Figure 6:**
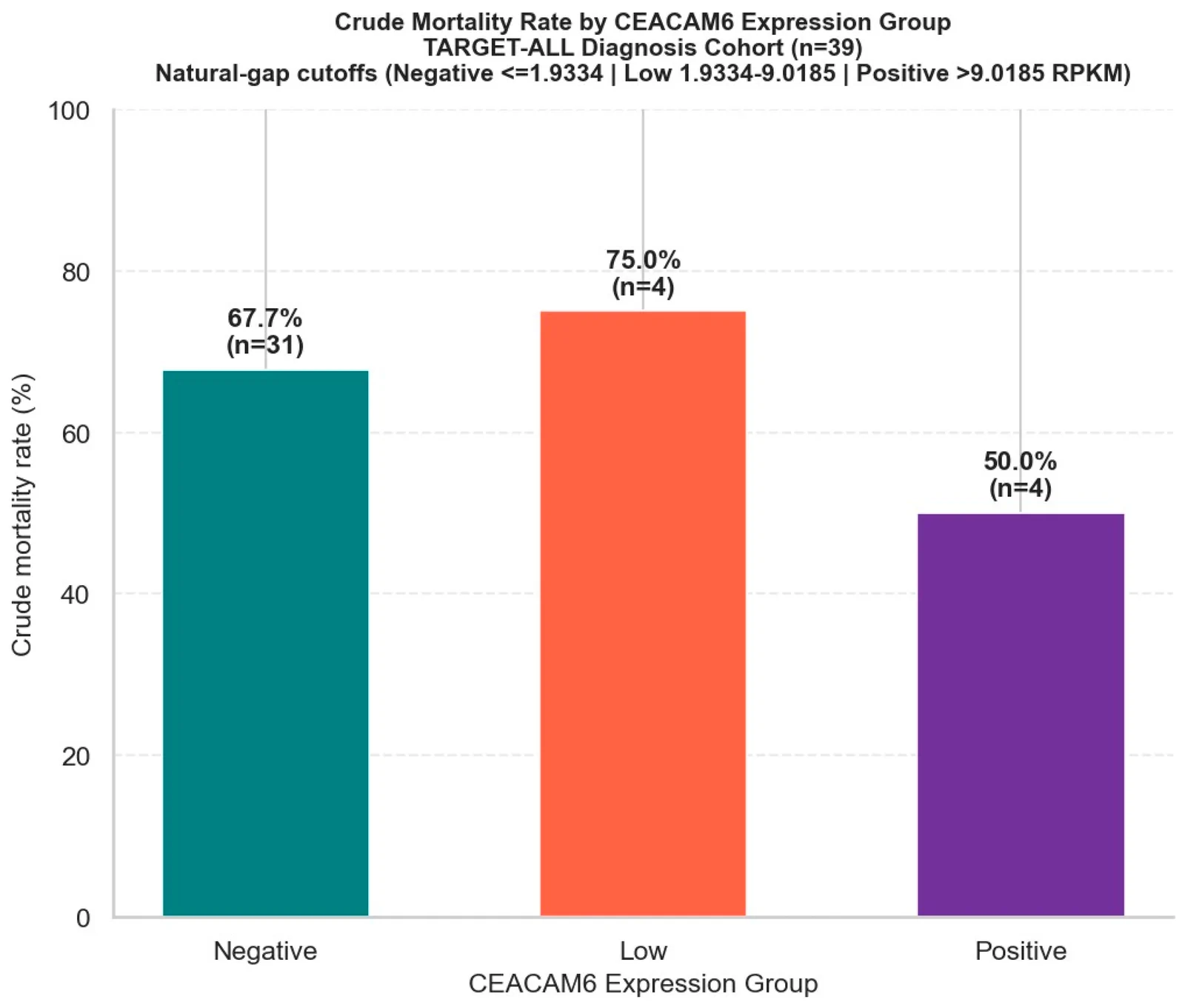
**Crude mortality rate by CEACAM6 expression group in the TARGET-ALL Phase II diagnosis cohort (*n* = 39)**. CEACAM6 RNA-seq expression (RPKM) was stratified into three descriptive groups using a natural-gap approach applied to the ordered distribution of diagnosis-stage samples, without reference to clinical outcomes: Negative (RPKM ≤ 1.9334, *n* =31), Low (1.9334 < RPKM ≤ 9.0185, *n* = 4), and Positive (RPKM > 9.0185, *n* = 4). The Negative/Low boundary corresponds to the largest gap among values below 5 RPKM (1.2232 units); the Low/Positive boundary corresponds to the second-largest gap in the full ordered distribution (4.6782 units). Bars represent the percentage of patients deceased at last follow-up (OS_STATUS). The CD66c^low^ group showed the highest crude mortality rate (75.0%), followed by Negative (67.7%) and Positive (50.0%). Pairwise Fisher’s exact test *p*-values: Negative vs. Low, *p* = 1.0000; Low vs. Positive, *p* = 1.0000; Negative vs. Positive, *p* = 0.5941. No statistically significant difference was observed between groups, consistent with the limited sample sizes.

To provide a preliminary transcriptomic context for the clinical findings observed in our Mexican pediatric cohort (Figure 1, Figure 2, Figure 3, Figure 4 and Figure 5), we performed an exploratory descriptive analysis of CEACAM6/CD66c RNA-seq expression (RPKM) in the TARGET-ALL Phase II public database, restricted to diagnosis-stage samples. Expression thresholds were derived using a natural-gap approach: the largest absolute discontinuities in the ordered RPKM distribution of the real diagnosis cohort were identified without reference to clinical outcomes, yielding three descriptive groups: Negative (RPKM ≤ 1.9334, *n* = 31), Low (1.9334 < RPKM ≤ 9.0185, *n* = 4), and Positive (RPKM > 9.0185, *n* = 4). Crude mortality rates (percentage of patients deceased at last follow-up, OS_STATUS) were calculated for each group. The CD66c^low^ group showed the highest crude mortality rate (75.0%), compared with 67.7% in the Negative group and 50.0% in the Positive group (Figure 6). Pairwise Fisher’s exact tests did not reach statistical significance for any comparison (Negative vs. Low: *p* = 1.0000; Low vs. Positive: *p* = 1.0000; Negative vs. Positive: *p* = 0.5941), consistent with the limited group sizes. Notwithstanding the absence of statistical significance, attributable to the small group sizes inherent to this stratification scheme, the directional pattern observed is consistent with the primary clinical findings of the present study: CD66c^low^ blasts are associated with a higher mortality burden in both our independent Mexican pediatric cohort and in this preliminary exploration of the TARGET-ALL transcriptomic data. This directional consistency supports the biological hypothesis proposed herein, while acknowledging that confirmation in larger cohorts with complete survival data is required.

## 4. Discussion

Dysregulated over-expression of CD66c is oncogenic and is associated with anoikis resistance and invasive phenotype mediated by excessive TGFβ, AKT, FAK and SRC signaling in human malignancies [10,11,15]. CEACAM6 modulates cancer progression through tissue destruction, aberrant cell differentiation, anti-apoptosis, cell growth, and resistance to therapeutic agents [16].

Here, we conducted, for the first time, a deeper analysis of aberrant CD66c expression in B-ALL pediatric patients. Within the hematopoietic system, CEACAM6 is expressed on the granulocytes and is involved in homotypic and heterotypic adhesion, as well as Ca2^+^-mediated signaling. Specifically, CEACAM6 increases the adhesiveness of the granulocytes to the extracellular matrix (ECM) components through activation of β1 and β2 integrins [17]. Importantly, CD66c appears to enhance apoptosis in blast cells, possibly through the activation of integrin signaling pathways [17]. Several studies have showed CEACAM6 is also aberrantly expressed in B-ALL [2,18,19] and this expression is associated with *BCR::ABL* translocation in MRD follow-up [20,21]; CD66c was also expressed in Ph-like kinase fusion-, *PAX5* fusion-, and *DUX4* fusion-positive ALL, but not in *MEF2D* fusion-positive ALL, indicating constant selectivity of CD66c expression [22]. In our cohort, we demonstrated that CD66c is the most prevalent aberrant antigen in B-ALL (Figure 1) and this expression was associated with increased detectable MRD and mortality when CD33 or CD123 were co-expressed. However, the expression itself marks different levels of this protein on B-ALL cell surfaces, which demonstrates that its expression can probably be modulated by extrinsic factors. Studies by Behrens et al. have demonstrated that, during chronic *H. pylori* infection, there is a systemic decrease in the expression of the hCEACAM1 and hCEACAM6 receptors on the surface of neutrophils [23]. This molecular attenuation likely functions as a strategic compensation by restricting the efficiency of CagA translocation mediated by the Type IV Secretion System 4 (TSS4), shifting the pathogen–host interface toward a state of attenuated inflammatory signaling. This regulatory mechanism also serves to mitigate acute gastric immunopathology, thereby fostering an environment conducive to long-term bacterial persistence and chronic colonization within the gastric niche.

In our cohort we demonstrated three levels of CD66c expression (Figure 1b). In line with other studies, leukemia cell lines had a uniform low expression of CEACAM family members [24]; our patients showed differences in CD66c expression among the ProB, ProB-PreB, and PreB subtypes (Figure 2) that indicate divergent biological states with potential prognostic implications. ProB leukemias most frequently showed positive expression and low levels of CD66c; ProB-PreB leukemias frequently presented a dichotomous pattern, positive expression or absence, and PreB leukemias exhibited minimal levels (Figure 2). This heterogeneity aligns with previous reports describing the myeloid restriction of CD66c in normal BM and its characteristic expression in early CD10^+^ B-cell malignancies, where its co-expression helps to distinguish residual leukemic blasts from normal regenerative populations [18]. This maturation-dependent gradient suggests that CD66c is not only an aberrant marker but also a reflection of early arrests in lymphoid maturation which could be associated with genetic determinants and extrinsic factors that promote worse prognoses in the Mexican pediatric population who are identified within Cluster 5, as previously reported by our group [2].

The immunotumoral profiles obtained by APS (Figure 2) clearly distinguish cases with low versus positive CD66c expression, pointing to early immune reconfigurations at disease onset. This observation is consistent with studies linking the microenvironment, inflammation, and alterations in pathways such as *PAX5*, IL-6, and Myd88 to leukemic evolution from preleukemic phases [25,26]. Recent multi-omics studies also show that dysregulation of regulatory programs in early B-cell lymphopoiesis defines transformation states associated with specific B-ALL subtypes [27], reinforcing the utility of phenotypic markers such as CD66c for identifying relevant biological transitions. Likewise, reviews by García et al. and Vilchis et al. highlight that the interaction between genetic alterations, inflammation, metabolism and microenvironment contributes to progression and therapeutic resistance in B-ALL [28,29], underscoring the importance of integrating immunophenotypic markers with extrinsic determinants of the disease.

From a functional perspective, the immunohematopoietic heterogeneity we describe could favor tumor survival strategies mediated by the leukemia–niche interaction and the CEACAM axis, contributing to a protective microenvironment that facilitates chemoresistance and immune evasion [26,27]. Analogous to the stealth mechanisms used by chronic pathogens to modulate innate responses, aberrant expression in low levels of CD66c in B-ALL subgroups could attenuate surveillance by NK cells and neutrophils, leading to differential tumor behaviors that influence clinical outcomes [23,30].

The immunosuppressive activity of CEACAM6 was mediated by binding to CEACAM1 expressed by activated tumor-specific T cells [31]. Furthermore, CEACAM6 has been shown to play an important role in suppressing CD8^+^ T cell responses to multiple myeloma, highlighting the pro-tumoral activity of this molecule in the tumor microenvironment [32].

CEACAM6 affects the tumor microenvironment (TME) through its close interactions with integrins and adhesion molecules. Antibody-mediated CEACAM6 cross-linking activates neutrophils and promotes their adhesion to the ECM. The cross-linking results in significantly increased attachment to fibronectin and vitronectin. High CEACAM6 expression shows a trend towards worse survival in HER2-overexpressing breast cancers [16]. In a study conducted to evaluate CD66c on PDA, after sample stratification according to high and low expression of CD66c, the researchers demonstrated a higher rate of low T-cell cytolytic activity, associating CD66c^high^ expression with immune suppression [33].

Pro-inflammatory cytokines induce and sustain the expression of CEACAM3 and CEACAM6 in intestinal epithelial cells (IECs). This was measured by treating IECs with a cocktail of cytokines including IL-1β, not only impacting CEACAM protein expression but also the induction of IL-8 [34].

Our findings reveal that the BM microenvironment in B-ALL is not a passive bystander but undergoes a profound reprogramming that could be linked to the blast’s immunophenotype. The observed correlation between CD66c expression and the upregulation of CXCL10, IL-1β, and NF-κB in MSCs suggests that CD66c^pos^ blasts may exert an ‘instructive’ role, converting the healthy hematopoietic niche into a pro-inflammatory malignancy-promoting niche. This is consistent with the paradigm that leukemic cells ‘hijack’ and remodel BM niches to favor their own survival while impairing normal hematopoiesis [35].

Notably, the microenvironmental divergence between CD66c^pos^- and CD66c^low^-groups, suggestively driven by CXCL10 (Figure 3a,b), highlights that the level of aberrant marker expression dictates the degree of stromal reconfiguration. The expression of LGALS9 and CD39 across CD66c^low-pos^ -groups is particularly striking. LGALS9 and CD39 are recognized immune checkpoints; while LGALS9 modulates T-cell response via the Tim-3 pathway [36,37], CD39 facilitates an immunosuppressive adenosine-rich environment [38]. This suggests that CD66c-associated niches are primed for immune evasion, a mechanism that could explain the therapeutic resilience observed in specific B-ALL clusters.

The regulatory mechanism demonstrated by Hu et al. in lung adenocarcinoma shows that the miR146a/CEACAM6 axis is a critical determinant of chemoresistance, where miR146a directly modulates CEACAM expression to influence susceptibility to cisplatin-based therapies [39]. In light of recent evidence showing miR146a overexpression [40] in B-ALL and its role in remodeling the proinflammatory microenvironment, we hypothesize that the CD66c^low^-phenotype observed in our Pro-B cohort represents a state of molecular stealth (Figure 4). From a clonal perspective, low CD66c expression may identify biologically aggressive or poorly differentiated subclones (Figure 4a) with enhanced survival capacity under therapeutic pressure. This aligns with the observed correlation between CD66c^low^ and adverse clinical outcomes.

Unlike the CD66c^high^ expression typically associated with specific genetic subgroups, this post-transcriptional downregulation mediated by miR146a^high^ may reduce the immunogenicity of the lymphoblasts. By maintaining a ‘low-profile’ immunophenotypic signature, these cells could evade residual immune surveillance while the BM niche is concurrently exhausted by pro-inflammatory factors driven by secreted miRNAs. Our data suggests that this miR146^high^/CD66c^low^ axis correlates with a shift toward stemness and a quiescent metabolic state, as evidenced by a slight increase in the CD34^+^ population within detectable MRD (Figure 4b, middle panel). Given that high CEACAM6 expression has been implicated in apoptosis resistance and the mobilization of CD34^+^ progenitors from the BM in myelofibrosis [41], its specific downregulation in our B-ALL model suggests a divergent but equally effective survival strategy: the preservation of a ‘stealth’ stem-like reservoir. This epigenetic editing of the CD34^+^ subpopulation potentially enhances niche retention and protects the leukemic seed from therapeutic elimination, providing a biological basis for the persistent MRD risk observed in vulnerable pediatric populations in Mexico.

Our findings reveal that low expression of CD66c in B-ALL and specifically in ProB blasts is significantly associated with poorer global survival and the presence of detectable MRD following induction therapy (Figure 5a–c). This association may be explained through several immunobiological and molecular mechanisms. As we discussed before, diminished CD66c levels may disrupt interactions between leukemic cells and the immune microenvironment, weakening immune surveillance and promoting the survival of resistant leukemic clones. This immune evasion could be particularly relevant in the context of MRD, where minimal disease burden escapes conventional detection and eradication.

The exploratory characterization of CEACAM6 transcriptomic expression in the TARGET-ALL Phase II public database (*n* = 39 diagnosis-stage patients) provides preliminary external context for the clinical findings reported in our Mexican pediatric cohort. Although the limited group sizes precluded a statistically powered survival analysis, the descriptive crude mortality pattern observed is directionally consistent with our primary findings: the CD66c^low^ group showed the highest crude mortality rate (75.0%), compared with 67.7% in the Negative group and 50.0% in the Positive group (Figure 6). This nonlinear tendency is aligned with our hypothesis that CD66c^low^ blasts may adopt a specialized, microenvironment-induced state of reduced immunogenicity that favors persistence under therapeutic pressure and higher MRD detectability. We acknowledge that these observations are exploratory and do not reach statistical significance in this cohort; confirmation in larger datasets with complete survival data is required to establish the prognostic value of *CEACAM6* transcriptomic expression as an independent determinant in pediatric B-ALL.

This complex prognostic behavior mirrors the ‘dual role’ of CEACAM6 reported in solid tumors. In gastric cancer, for instance, high CEACAM6 expression correlates with improved OS in early stages but shifts to a marker of poor prognosis in advanced disease [15]. Similarly, in pancreatic and colon cancers, the synergy between CEACAM6 and immune-modulating factors, such as FOXP3^+^ regulatory T cells [42], underscores its role in orchestrating a suppressive immune landscape that drives progression [43].

By integrating these observations, we propose that in Pro-B ALL, the low CD66c phenotype represents an evolutionary adaptive advantage for the tumor. Similar to stage-dependent change in gastric cancer, this low-level induction could reflect a transition to a latent, chemoresistant, and immunoevasive state. By avoiding high-level expression that could trigger greater immune recognition, these blasts maintain sufficient CEACAM6-mediated signaling to survive within the protective niche of the BM. Consequently, the impact of comparing CD66c levels in our cohort validates the hypothesis that Pro-B ALL blasts with low CD66c possess greater microenvironment-induced persistence capacity, which directly translates into the higher mortality rates observed in vulnerable pediatric populations. Limitations of our study include the limited availability of MRD data, which were obtained from only 50 patients in the cohort, and the fact that the median interval of MRD assessment was 56 days (range, 24–105). This wide range was primarily due to infections or other adverse events affecting the patients. More extensive clinical data may help strengthen the association. Further studies have to be performed to confirm our hypothesis, focusing on dissecting the functional roles of immune cells in B-ALL with aberrant CD66c expression and the impact on BM microenvironment; some of the approaches include determining pro- or anti-tumoral activities of immune cells, like NK cells tumoral modulation (previously reported by our group [44]); blocking the interaction of the CEACAM6 binding of B-ALL blasts with CEACAM receptors in the microenvironment cells; assessing CD8^+^ T-cell possible suppression in CD66c^low^ patients, which could clarify additional immune evasion mechanisms. Experiments on mesenchymal stromal cells exposed to CD66c^low^ blasts, including 3D cultures, will help determine stromal reconfiguration and chemoresistance. Moreover, evaluating whether miR146 downregulates CD66c in CD66chi blasts may uncover novel regulatory pathways.

## 5. Conclusions

Our study redefines the presence of CD66c not only as an aberrant diagnostic marker but also as a functional orchestrator of the leukemia–niche interaction in pediatric B-ALL. We have demonstrated maturation-dependent expression (ProB leukemias) where the intensity of CD66c expression, rather than its mere presence, dictates clinical prognoses. Specifically, the CD66c^low^ phenotype in ProB-ALL represents a critical adaptation optimum that correlates with a significantly higher risk of MRD detectability and mortality. Our findings provide strong evidence of bidirectional BM niche remodeling. We propose that CD66c^low^ blasts, likely regulated by miR146, adopt a “molecular hiding” or “silent state” strategy. This allows them to evade immune surveillance, which is associated with the overexpression of checkpoints such as LGALS9 and CD39 in the BM niche, enabling their low immunogenic latency and promoting chemoresistance and long-term persistence. Ultimately, the validation of this CD66c intensity-dependent risk using the TARGET-ALL database underscores the urgent need to integrate multiparametric immunophenotypic substratification into clinical practice. Further experimental work is needed to validate our conclusions. Identifying these hidden reservoirs at the time of diagnosis is essential for refining prognosis and developing precision interventions, particularly for vulnerable pediatric populations in Mexico, where socio-environmental stressors can exacerbate this aggressive biological reconfiguration.

## Figures and Tables

**Table 1:** **Patient cohort characteristics.**

Characteristics	*n* (%)
B-ALL patients	Total 128 (100)
Male	66 (51.56)
Mean age	8.15 years
ProB-ALL	65 (50.78)
ProB-PreB-ALL	44 (34.38)
PreB-ALL	19 (14.84)
Non-AL patients	Total 4 (100)
Male	4 (100)
Mean age	2.88 years
Healthy children (controls)	Total 6 (100)
Male	5 (83.33)
Mean age	12.83 years

## Data Availability

The datasets generated and/or analyzed during the current study are not publicly available due to patient confidentiality and ethical restrictions. Although anonymized identifiers (acronyms) were used, the underlying clinical data cannot be shared to protect participant privacy.

## Supplementary Materials

The following supporting information can be downloaded at: https://www.mdpi.com/article/10.3390/cells15050437/s1, Figure S1: **Determination of aberrant myeloid antigens expression.** CD33, CD15, CD123 and CD13 expression levels were classified as positive, low or negative based on the comparison of mean fluorescence intensity (MFI) between blast cells of B-ALL and neutrophils, monocytes or CD34^+^ HSPC cells as positive control, and lymphocytes as a negative control; Figure S2: **Mesenchymal Stromal Cell Microenvironment in ProB-ALL related to CD66c expression.** Evaluation of inflammatory and suppressive profiles in MSC derived from patients with ProB-ALL. (a) Phenotypic assessment of MSCs obtained at disease diagnosis (*n* = 12) according to the presence or absence of blasts with aberrant CD66c expression. (b) Phenotypic evaluation of MSCs obtained during disease follow-up (*n* = 7). CXCL, C-X-C motif chemokine ligand; LGALS9, galectin-9; MFI, Median Fluorescence Intensity; * *p* < 0.05. Graphs include violin plots and box-and-whisker plots, dotted lines in violin plots represent medians of groups; Table S1: **Distribution of B-ALL cases and monitoring of treatment response based on CD66c expression levels.** CD66c expression levels were categorized as positive, low or negative based on the comparison of the mean fluorescence intensity (MFI) between blast cells and neutrophils, which served as a positive control for expression. Relative risk (RR) values were calculated based on B-ALL CD66c expression and B-ALL subtype (each subgroup compared to CD66c-negative expression).

## Abbreviations

The following abbreviations are used in this manuscript:

AKT	Protein Kinase B
AL	Acute Leukemia
ALL	Acute Lymphoblastic Leukemia
ALOT	Acute Leukemia Orientation Tube
APS	Automatic Population Separator
B-ALL	B-cell precursor acute lymphoblastic leukemia
BM	Bone marrow
CEA	Carcinoembryonic antigen
CEACAM3	Carcinoembryonic antigen-related cell adhesion molecule 3
CEACAM6	Carcinoembryonic antigen-related cell adhesion molecule 6
CIBIOR	Centro de Investigación Biomédica de Oriente
CI	Confidence interval
CXCL8	C-X-C motif chemokine ligand 8
CXCL10	C-X-C motif chemokine ligand 10
EOI	End of induction therapy
ECM	Extracellular matrix
FAK	Focal Adhesion Kinase
FOXP3	Forkhead Box P3
GPI	Glycosylated glycosylphosphatidylinositol
IECs	Intestinal epithelial cells
IMSS	Instituto Mexicano del Seguro Social
IL-1β	Interleukin 1-beta
IL-6	Interleukin 6
LGALS9	Galectin-9
MRD-HR	High-risk Measurable Residual Disease
MRD-LR	Low-risk Measurable Residual Disease
MFI	Mean fluorescence intensity
miR146a	microRNA-146a
miRNAs	microRNAs
MSCs	Mesenchymal Stromal Cells
MRD	Measurable Residual Disease
NF-κB	Nuclear factor-kappa B
OS	Overall Survival
PDA	Pancreatic ductal adenocarcinoma
PFS	Progression-Free Survival
RR_MRDd_	Relative risk values of detectable MRD
SECIHTI	Secretaría de Ciencia, Humanidades, Tecnología e Innovación
SD	Standard deviation
SRC	Proto-oncogene Tyrosine-protein Kinase
TGFβ	Transforming Growth Factor-beta
TME	Tumor microenvironment
TSS4	Type IV Secretion System 4
UMAP	Uniform Manifold Approximation and Projection
