# Correction: Zamora-Herrera et al. Low-Intensity CD66c Expression Orchestrates an Immunosuppressive Niche Promoting Residual Disease in Pediatric ProB Acute Lymphoblastic Leukemia. *Cells* 2026, *15*, 437

**DOI:** 10.3390/cells15211968

**Published:** 2026-10-29

**Authors:** Gabriela Zamora-Herrera, Rubí Romo-Rodríguez, Jebea A. López-Blanco, Laura Alfaro-Hernández, Diana Casique-Aguirre, Juan Carlos Núñez-Enríquez, Michael Schnoor, Dalia Ramírez-Ramírez, Rosana Pelayo

**Affiliations:** 1 Laboratory of Oncoimmunology and Cytomics of Childhood Cancer, Centro de Investigación Biomédica de Oriente, Instituto Mexicano del Seguro Social, Puebla 74360, Mexico; gabriela.zamorah@alumno.buap.mx (G.Z.-H.); rubi.romo@imss.gob.mx (R.R.-R.); jebea22@yahoo.com.mx (J.A.L.-B.); laura.alfarohernandez@viep.com.mx (L.A.-H.); dianacasique@gmail.com (D.C.-A.); 2 Instituto de Fisiología, Benemérita Universidad Autónoma de Puebla, Puebla 72570, Mexico; 3 Secretaría de Ciencia, Humanidades, Tecnología e Innovación (Secihti), Mexico City 03940, Mexico; 4 Facultad de Ciencias Químicas, Benemérita Universidad Autónoma de Puebla, Puebla 72570, Mexico; 5 División de Investigación en Salud, Unidad Médica de Alta Especialidad (UMAE) Hospital de Pediatría “Silvestre Frenk Freund” Centro Médico Nacional Siglo XXI, Instituto Mexicano del Seguro Social, Mexico City 06720, Mexico; jcarlos_nu@hotmail.com; 6 Department of Molecular Biomedicine, Cinvestav-IPN, Mexico City 07360, Mexico; mschnoor@cinvestav.mx; 7 Unidad de Educación e Investigación, Instituto Mexicano del Seguro Social, Mexico City 06725, Mexico

## Error in Figure

In the original publication [1], there was a mistake in the legend for Figure 6. The qualitative CEACAM6 expression thresholds (Negative, Low, Positive) applied to the TARGET-ALL Phase II cohort were derived from a visual assessment of the expression distribution rather than from an explicitly defined, statistical criterion, and the restriction of the analysis to diagnosis-stage samples (GDC sample type code “−03”, *n* = 39) was not stated. To improve the original stratification and eliminate any potential ambiguity, an unsupervised, outcome-blind manner was performed. The correct legend appears below. 

In the original publication [1], there was a mistake in Figure 6 as published. In the original figure, a visual stratification method based on the distribution of expression was employed, and the total number of patients in the database was analyzed (Negative *n* = 23, Low *n* = 46, Positive *n* = 129). To improve stratification, validity, and reproducibility of the data, patients were re-stratified using a natural-gap approach applied to the distribution of the data, considering only those patients at diagnosis within the cohort (*n* = 39). Given that original OS_MONTHS data was available for only 16 of 39 patients and thus preclude a well-powered time-to-event analysis across three expression groups; crude mortality rate was preferred for the analysis since OS_STATUS information was complete. The corrected Figure 6, reflecting the correct classification (Negative *n* = 31, Low *n* = 4, Positive *n* = 4), appears below.

**Figure 6:**
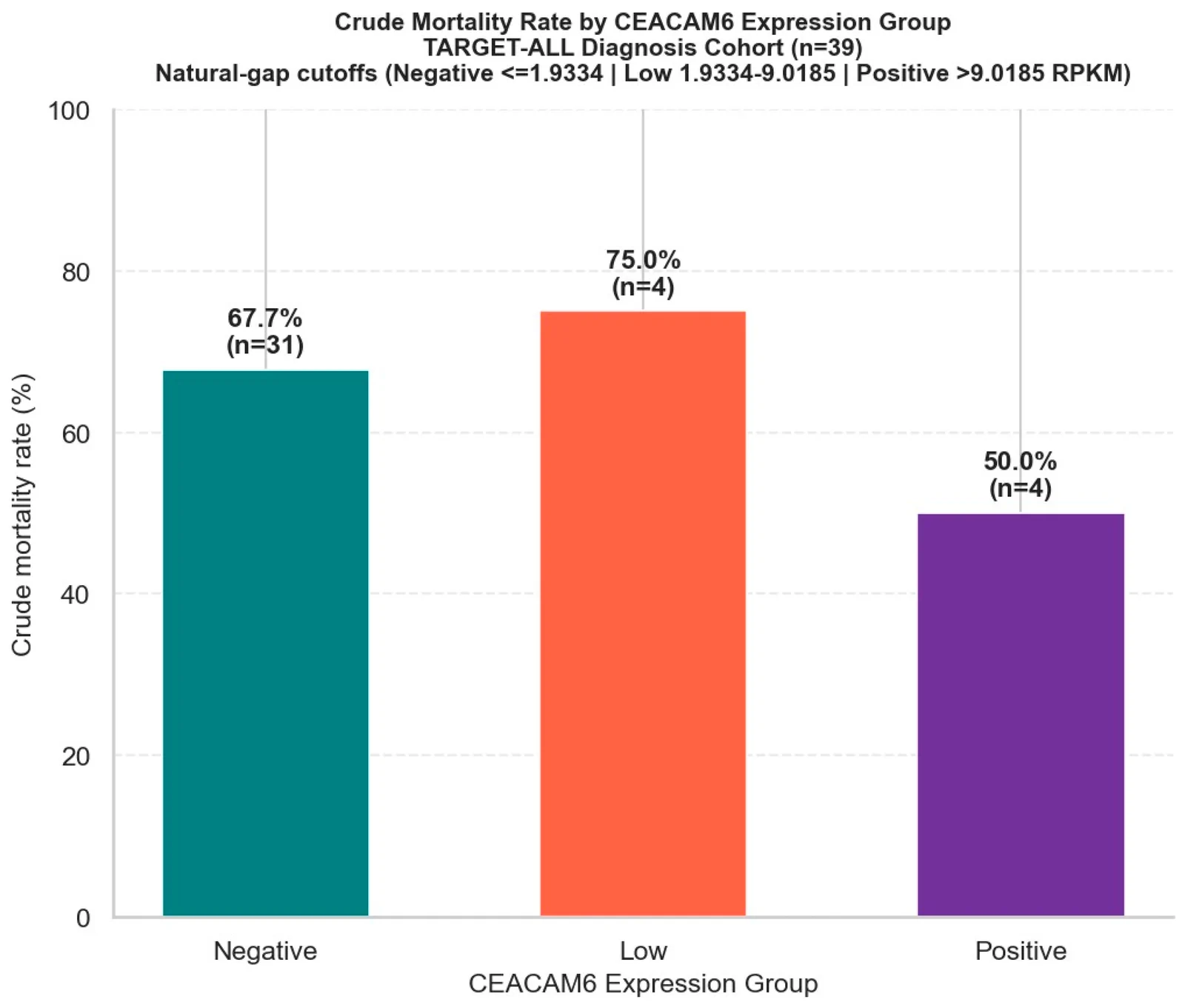
**Crude mortality rate by CEACAM6 expression group in the TARGET-ALL Phase II diagnosis cohort (*n* = 39)**. CEACAM6 RNA-seq expression (RPKM) was stratified into three descriptive groups using a natural-gap approach applied to the ordered distribution of diagnosis-stage samples, without reference to clinical outcomes: Negative (RPKM ≤ 1.9334, *n* =31), Low (1.9334 < RPKM ≤ 9.0185, *n* = 4), and Positive (RPKM > 9.0185, *n* = 4). The Negative/Low boundary corresponds to the largest gap among values below 5 RPKM (1.2232 units); the Low/Positive boundary corresponds to the second-largest gap in the full ordered distribution (4.6782 units). Bars represent the percentage of patients deceased at last follow-up (OS_STATUS). The CD66c^low^ group showed the highest crude mortality rate (75.0%), followed by Negative (67.7%) and Positive (50.0%). Pairwise Fisher’s exact test *p*-values: Negative vs. Low, *p* = 1.0000; Low vs. Positive, *p* = 1.0000; Negative vs. Positive, *p* = 0.5941. No statistically significant difference was observed between groups, consistent with the limited sample sizes.

## Text Correction

There was an error in the original publication [1]. The Materials and Methods Section did not fully specify the statistical criterion used to define the CEACAM6 expression thresholds (Negative, Low, Positive) applied to the TARGET-ALL Phase II RNA-seq cohort, nor did it state that the analysis was restricted to diagnosis-stage samples. As a consequence, the corresponding descriptive statistics reported in the Results Section have also been revised for consistency. This information has been added below for full methodological transparency.

A correction has been made to Materials and Methods, Section 2.7:

*2.7. Crude Mortality Analysis by CEACAM6 Expression Group in the TARGET-ALL Phase II Cohort*

RNA-Seq gene expression data (RPKM) and clinical variables from pediatric patients with B-ALL were obtained from the TARGET-ALL Phase II project. CEACAM6 expression data were retrieved from the TOIL/XenaBrowser repository, while clinical data was sourced from the GDC Data Portal. Only samples obtained at the time of initial diagnosis (GDC sample type code “−03”) were included in this analysis (*n* = 39 patients with complete expression and vital status data), thereby excluding recurrent tumor samples (“−04”), blood-derived normal/germline control samples (“−09/−10”), and recurrent peripheral blood samples (“−40”). Crude mortality (vital status at last follow-up, OS_STATUS, available for all 39 patients) was used as the primary descriptive outcome, since overall survival in months (OS_MONTHS) was available for only 16 of the 39 patients. CEACAM6 expression was stratified into three descriptive groups using a natural-gap approach: expression thresholds were defined from empirically observed discontinuities in the ordered RPKM distribution of the 39 diagnosis-stage patients, identified without reference to clinical outcomes. The Negative/Low boundary corresponds to the largest gap among values below 5 RPKM (gap = 1.2232 RPKM units, located between 1.9334 and 3.1566 RPKM), and the Low/Positive boundary corresponds to the second-largest gap in the full ordered distribution (gap = 4.6782 RPKM units, located between 4.3403 and 9.0185 RPKM), yielding Negative (RPKM ≤ 1.9334, *n* = 31), Low (1.9334 < RPKM ≤ 9.0185, *n* = 4), and Positive (RPKM > 9.0185, *n* = 4) groups. As a pre-specified sensitivity analysis, an unsupervised equal-size (tertile) stratification and Jenks natural breaks of the same cohort were also examined.

A correction has been made to Materials and Methods, Section 2.8

*2.8. Statistical Analysis of Crude Mortality*

Crude mortality rates (percentage of patients deceased at last follow-up) were calculated for each CEACAM6 expression group (Negative, Low, Positive) as defined in Section 2.7. Pairwise comparisons of crude mortality rates between groups were performed using Fisher’s exact test. All analyses were implemented in Python 3.11 using pandas, numpy, scipy, matplotlib, and seaborn. Bar charts were generated with standardized parameters.

A correction has been made to the Results Section, Section 3.8.

*3.8. CEACAM6 Expression in the TARGET-ALL Phase II Cohort: A Preliminary Exploratory Characterization of Crude Mortality*

To provide a preliminary transcriptomic context for the clinical findings observed in our Mexican pediatric cohort (Figures 1–5), we performed an exploratory descriptive analysis of CEACAM6/CD66c RNA-seq expression (RPKM) in the TARGET-ALL Phase II public database, restricted to diagnosis-stage samples. Expression thresholds were derived using a natural-gap approach: the largest absolute discontinuities in the ordered RPKM distribution of the real diagnosis cohort were identified without reference to clinical outcomes, yielding three descriptive groups: Negative (RPKM ≤ 1.9334, *n* = 31), Low (1.9334 < RPKM ≤ 9.0185, *n* = 4), and Positive (RPKM > 9.0185, *n* = 4). Crude mortality rates (percentage of patients deceased at last follow-up, OS_STATUS) were calculated for each group. The CD66c^low^ group showed the highest crude mortality rate (75.0%), compared with 67.7% in the Negative group and 50.0% in the Positive group (Figure 6). Pairwise Fisher’s exact tests did not reach statistical significance for any comparison (Negative vs. Low: *p* = 1.0000; Low vs. Positive: *p* = 1.0000; Negative vs. Positive: *p* = 0.5941), consistent with the limited group sizes. Notwithstanding the absence of statistical significance, attributable to the small group sizes inherent to this stratification scheme, the directional pattern observed is consistent with the primary clinical findings of the present study: CD66c^low^ blasts are associated with a higher mortality burden in both our independent Mexican pediatric cohort and in this preliminary exploration of the TARGET-ALL transcriptomic data. This directional consistency supports the biological hypothesis proposed herein, while acknowledging that confirmation in larger cohorts with complete survival data is required.

A correction has been made to the Discussion Section, paragraph 14.

The exploratory characterization of CEACAM6 transcriptomic expression in the TARGET-ALL Phase II public database (*n* = 39 diagnosis-stage patients) provides preliminary external context for the clinical findings reported in our Mexican pediatric cohort. Although the limited group sizes precluded a statistically powered survival analysis, the descriptive crude mortality pattern observed is directionally consistent with our primary findings: the CD66c^low^ group showed the highest crude mortality rate (75.0%), compared with 67.7% in the Negative group and 50.0% in the Positive group (Figure 6). This nonlinear tendency is aligned with our hypothesis that CD66c^low^ blasts may adopt a specialized, microenvironment-induced state of reduced immunogenicity that favors persistence under therapeutic pressure and higher MRD detectability. We acknowledge that these observations are exploratory and do not reach statistical significance in this cohort; confirmation in larger datasets with complete survival data is required to establish the prognostic value of *CEACAM6* transcriptomic expression as an independent determinant in pediatric B-ALL.

The authors state that the scientific conclusions are unaffected. This correction was approved by the Academic Editor. The original publication has also been updated.
