# Supplementary figures and images for: Low-Intensity CD66c Expression Orchestrates an Immunosuppressive Niche Promoting Residual Disease in Pediatric ProB Acute Lymphoblastic Leukemia
Source: Cells. 2026 Feb 28;15(5):437. doi: 10.3390/cells15050437 (PMC12984215; doi:10.3390/cells15050437)

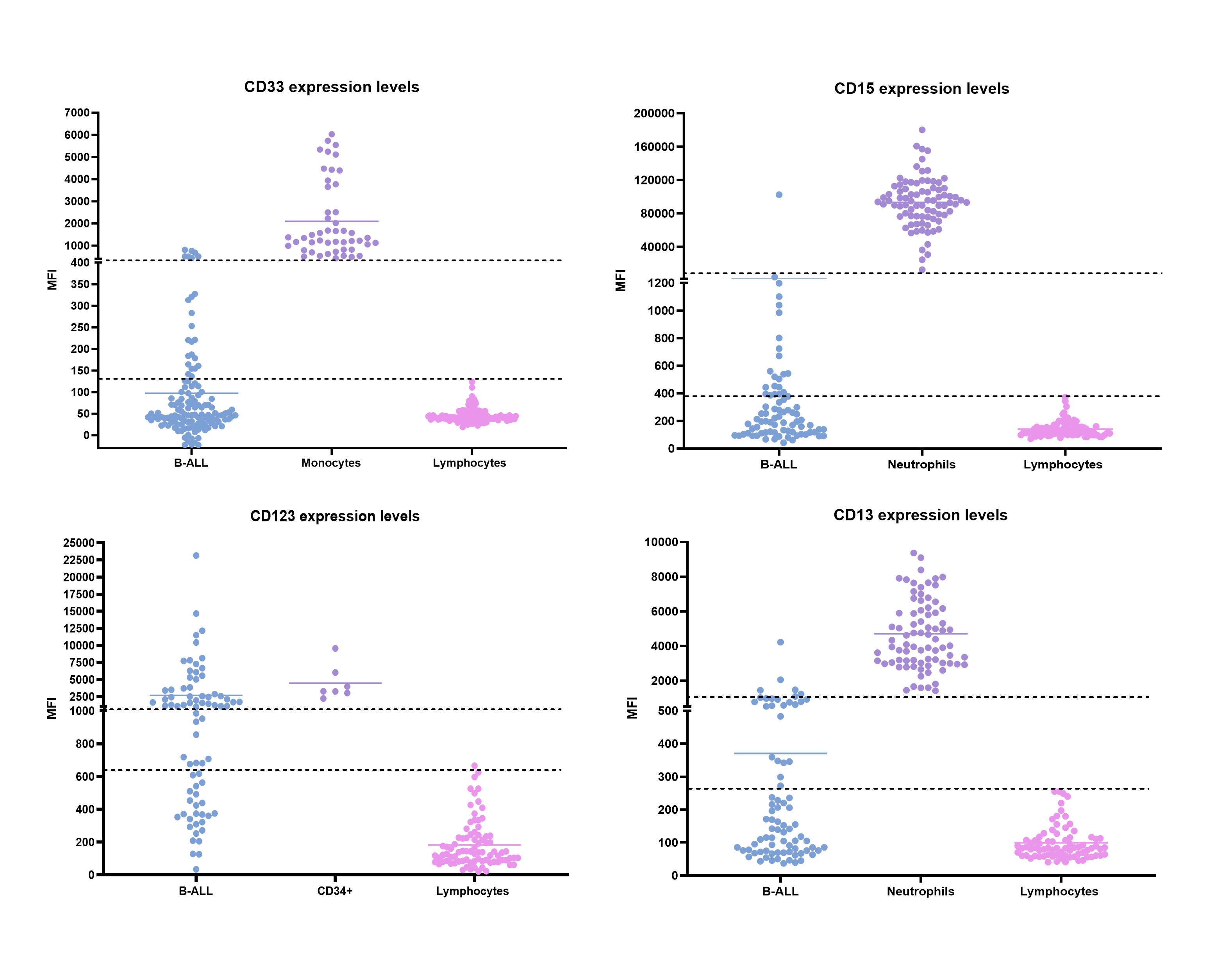

Supplement: Supplementary file 1 [file cells-15-00437-s001.zip › Supplementary Figure S1. Determination of aberrant myeloid antigen expression.png]

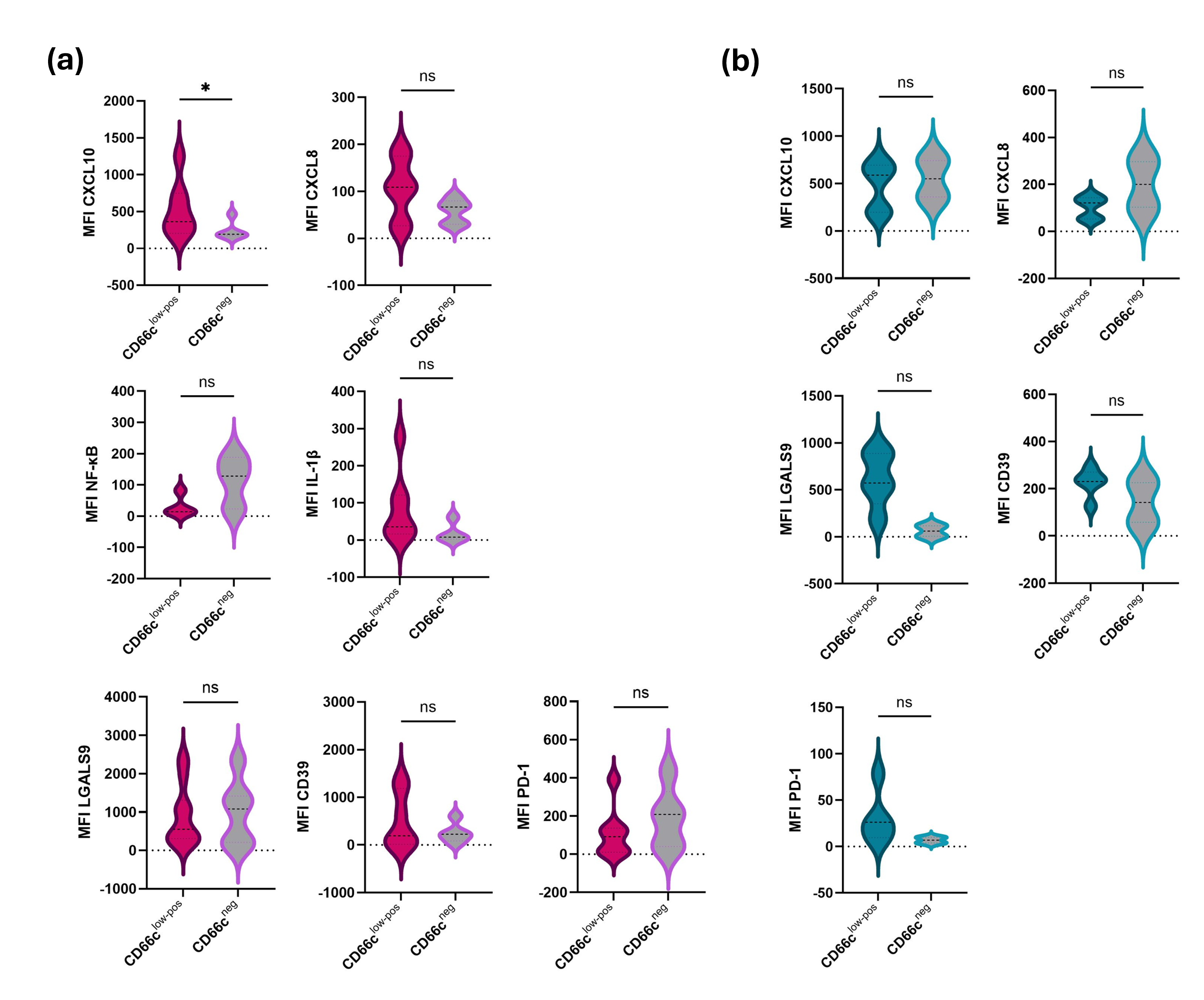

Supplement: Supplementary file 1 [file cells-15-00437-s001.zip › Supplementary Figure S2. Mesenchymal Stromal Cell Microenvironment in ProB-ALL related to CD66c expression.png]

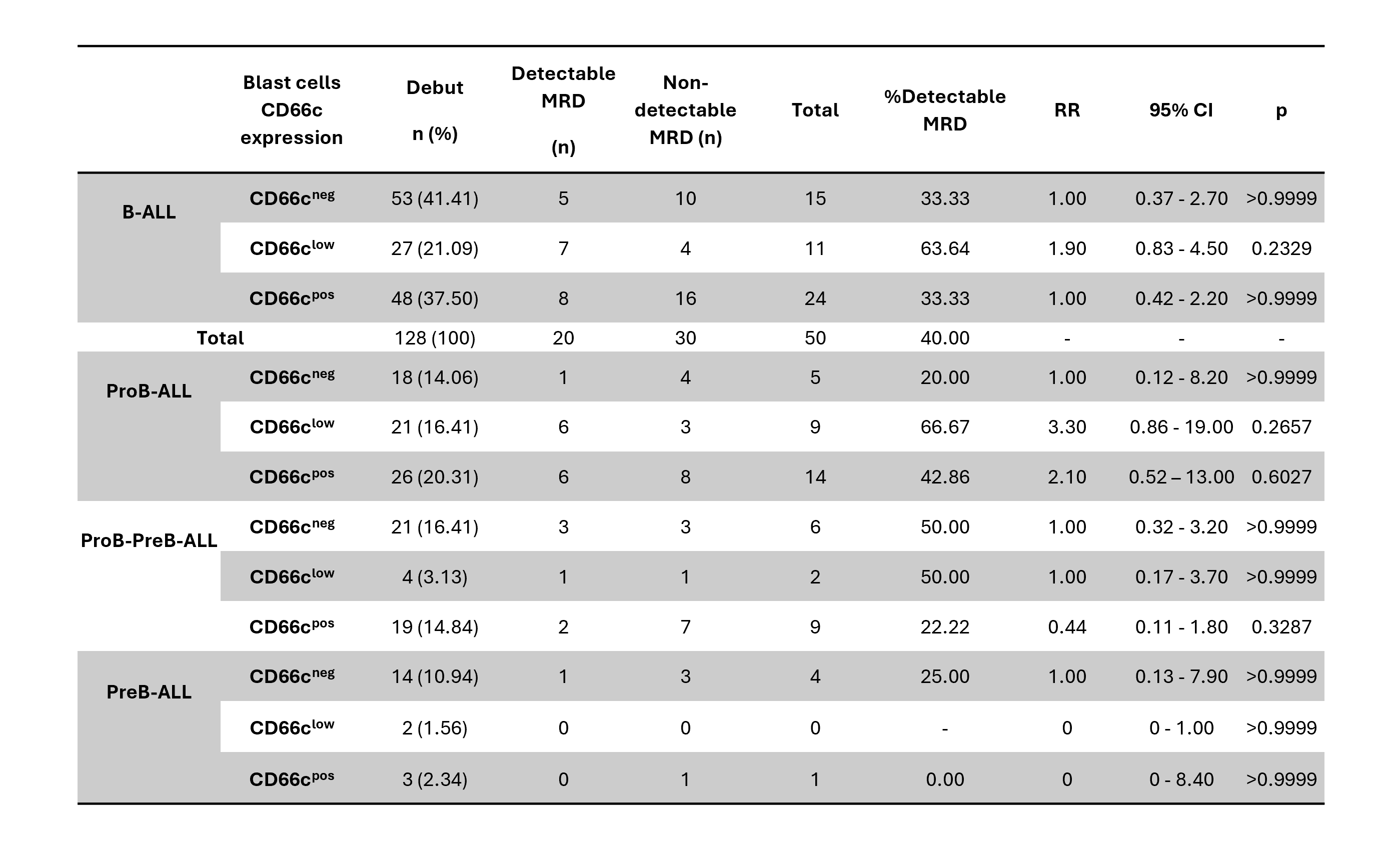

Supplement: Supplementary file 1 [file cells-15-00437-s001.zip › Supplemetary Table S1. Distribution of B-ALL cases and monitoring of treatment response based on CD66c expression levels.png]
